# Heavy Menstrual Bleeding-Visual Analog Scale, an Easy-to-Use Tool for Excessive Menstrual Blood Loss That Interferes with Quality-of-Life Screening in Clinical Practice

**DOI:** 10.1089/whr.2021.0139

**Published:** 2022-05-09

**Authors:** Josep Perelló, Pau Pujol, Montse Pérez, Maite Artés, Joaquim Calaf

**Affiliations:** ^1^ Department of Obstetrics and Gynecology, Hospital de Sant Pau, Barcelona, Spain.; ^2^ Universitat Autònoma de Barcelona, Barcelona, Spain.; ^3^ Bayer Hispania, Barcelona, Spain.; ^4^ Adelphi Targis, Barcelona, Spain.

**Keywords:** heavy menstrual bleeding, excessive menstrual blood loss, screening tool, visual analog scale, VAS, quality of life

## Abstract

**Background:**

Heavy menstrual bleeding (HMB) is defined as excessive menstrual blood loss that interferes with quality of life (QoL). The methods for assessing HMB are not suited for clinical practice. We analyzed the validity of a combined visual analog scale (VAS) tool assessing the intensity of menstrual bleeding (VASInt) and its impact on activities of daily living (VASImp) to identify women with HMB.

**Materials and Methods:**

Analysis conducted in the data set used to validate the Spanish HMB screening tool SAMANTA questionnaire. A logistic regression analysis was used to construct the model. Reference standard was the pictorial blood loss assessment chart (PBAC). The performance of the HMB-VAS and the SAMANTA questionnaire was compared. Correlation with SAMANTA questionnaire, PBAC, and other QoL measurements was assessed.

**Results:**

The resulting function (HMB-VAS score = 10.86 × VASInt score +2.48 × VASImp score) showed a slightly lower accuracy versus the SAMANTA questionnaire (86.8% vs. 87.9%) but a similar area under the curve: 0.9396 versus 0.943, respectively (*p* = 0.6605). The cutoff point was established as 700. After rounding the regression coefficients, the resulting function (11 × VASInt +2 × VASImp) showed 87.6% accuracy. The correlation of HMB-VAS with the SAMANTA questionnaire was strong (*r*: 0.79819; *p* < 0.0001), whereas the correlation was moderate to strong with the PBAC (0.59299; *p* < 0.0001) and weak with the QoL (EuroQoL five dimensions five levels questionnaire [EQ-5D-5L]) and well-being (Psychological General Well-Being Index [PGWBI]) scales (EQ-5D-5L VAS and Index: −0.20332 and −0.24384; PGWBI: −0.21680; *p* < 0.0001 for both).

**Conclusion:**

The HMB-VAS shows good performance for HMB screening, providing an easy-to-use alternative to other psychometric tools.

## Introduction

Heavy menstrual bleeding (HMB), which has been arbitrarily defined as a blood loss of 80 mL or more,^1,2^ is a common gynecological problem in clinical practice^3^ and a major cause of gynecological morbidity.^4^ The International Federation of Gynecology and Obstetrics (FIGO) defined abnormal uterine bleeding as “bleeding that is abnormally heavy and/or abnormal in timing” and HMB as “bleeding above the 95th percentile of the normal population.”^5^

In 2018, the more patient-centered definition proposed by the National Institute for Health and Care Excellence (NICE) was also adopted by FIGO for clinical purposes,^6^ this being “excessive menstrual blood loss (EML) which interferes with a woman's physical, social, emotional and/or material quality of life (QoL).”^3^ Besides the distress caused by the blood loss itself, HMB frequently results in iron deficiency anemia,^7^ which is responsible for symptoms such as fatigue, weakness, and dizziness that further undermine the woman's QoL.^8^ Moreover, HMB may be the presenting symptom of bleeding disorders.^9^ Suspicion, screening, and adequate diagnosis of HMB are therefore of utmost importance.

Numerous methods have been developed for quantifying menstrual blood loss.^10^ Although quantitative methods to assess HMB such as the objective alkaline hematin method^11^ remain important in the research setting and to objectively assess the treatment response, their use is challenging in clinical practice. The semiquantitative pictorial blood loss assessment chart (PBAC), where women provide a self-assessment of their monthly loss,^10^ has proved to be more accurate than subjective assessment alone.^12^ As originally developed by Higham et al., a PBAC score of >100 has shown a specificity and sensitivity of >80% when used as a diagnostic test for HMB.^13^

A recent systematic review of the value of PBACs to assess HMB has concluded that these methods “are best suited to the controlled and specific environment of clinical studies, where clinical outcome parameters are defined.” Moreover, the current lack of standardization precludes their widespread use in clinical practice.^14^ Tools assessing the effect of excessive menstrual blood loss (EML) on QoL and well-being, such as the Menorrhagia Multi-Attribute Scale (MMAS), have also been developed.^15^ Despite the usefulness of these tools in clinical practice, they are time-consuming for patients and practitioners, which is especially relevant in settings where patient consultation time is limited.

Recently, a six-item questionnaire (SAMANTA) has been developed in Spain with the aim of providing an easy screening tool to identify women with HMB (*i.e.*, with EML that interferes with QoL) in clinical practice.^16^ This tool, addressing both quantitative and qualitative aspects of HMB, has shown a sensitivity of 86.7% and a specificity of 89.5% to classify women as with PBAC-confirmed HMB or without it. Unfortunately, it has not been validated in other languages or cultural environments.

In the validity analysis, a strong correlation was found between the SAMANTA questionnaire score and the visual analog scale (VAS) scores for the intensity of menstrual bleeding (VASInt) and for its impact on activities of daily living (VASImp) (*p* < 0.001). The easy-to-use VAS scale has proven to be useful regardless of users' literacy level^17^ and allows cross-cultural adaptations with minimal translation difficulties.^18^ We analyzed the validity of using both VAS measurements in a single screening tool (hereafter termed HMB-VAS) in clinical practice.

## Materials and Methods

### Data source

Our analysis was conducted in the data set used to validate the Spanish SAMANTA questionnaire. The design of the psychometric validation study has been described elsewhere.^16^ Briefly, it was multicenter cross-sectional study in consecutive women aged ≥18 years seen at 11 Spanish hospitals who were at childbearing age. HMB cases consisted of women presenting HMB as confirmed by PBAC within the previous 3 months. Alternatively, women with a high clinical probability of HMB who were willing to complete the PBAC method during their next menstruation or women in whom treatment for HMB had to be started immediately according to the practitioner's criterion were also included in the group of women with HMB.

A control group of women who had completed the PBAC but did not meet the above-mentioned criteria and were not being treated with anticoagulants was also included. The PBAC was used as described by Higham et al.^13^ A PBAC score of ≥100 was used as the threshold on the basis of its diagnostic value as described in the original publication (sensitivity and specificity of 86.7% and 89.5% to classify women as with PBAC-confirmed HMB or without it, respectively).^13^

The exclusion criteria included amenorrhea or menopause, current use of hormonal contraception (including a hormonal intrauterine system) or use of a copper intrauterine device, or a history of malignancy or degenerative disease. Women who had undergone a hysterectomy or who had given birth within the previous 6 months were also excluded from participating.

The study population consisted of 211 women with PBAC-confirmed HMB and 153 women without it (controls), whose sociodemographic and clinical characteristics have already been published.^16^ Briefly, the mean age was 37.5 ± 8.9 years for women with HMB and 32.4 ± 8.0 years for women without HMB (*p* < 0.001); overall, 89.3% were Spanish. Sixty-three percent versus 41.2% presented dysmenorrhea, respectively (*p* < 0.0001), and 16.1% versus 5.3% presented intermenstrual bleeding (*p* = 0.0014). Eighty percent versus 0% had reported having HMB.

The frequency of menstrual bleeding was more frequently <21 days in women with HMB (14.2% vs. 3.9%; *p* = 0.0418). Menstrual bleeding was considered “abundant” in 100% of women with HMB while “normal” or “scarce” in 82.4% and 17.6%, respectively, of women without HMB (*p* < 0.0001). The duration of menstrual bleeding was longer (>7 days in 22.7% of women with HMB vs. 0% in the control group). The mean number of pregnancies and live deliveries was also higher in women with HMB (1.3 ± 1.4 vs. 0.7 ± 1.0 and 1.8 ± 0.8 vs. 1.4 ± 0.8).

All the participants provided written informed consent before being enrolled in the study. The study was approved by the ethics committee of Hospital de la Santa Creu i Sant Pau (Barcelona, Spain). The study was conducted in accordance with the standards of good clinical practice and the current version of the Declaration of Helsinki.

### Data collection and assessments

Sociodemographic and clinical data were recorded during a single routine clinical visit, as described elsewhere.^16^ During this visit, the PBAC was recorded when available, as mentioned previously. PBAC score ranged from 3 to 358. Fifty-four women have a PBAC score between 80 and 120. Fourteen women lacked a PBAC assessment.

**FIG. 1.**
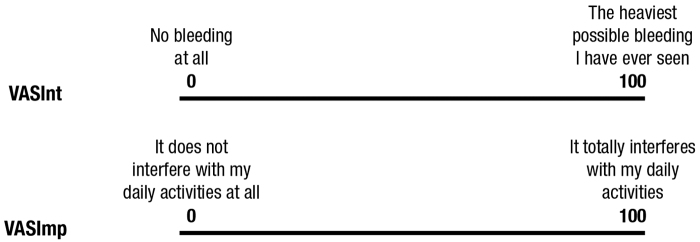
HMB-VAS construct to identify HMB. HMB, heavy menstrual bleeding; VAS, visual analog scale; VASInt, VAS measuring intensity of menstrual bleeding; VASImp, VAS measuring the impact of excessive menstrual blood loss on activities of daily living.

All assessments needed for the validation of the SAMANTA questionnaire took place during this single visit. Women were asked to complete a set of assessments, namely:

- The SAMANTA questionnaire, which is a six-item questionnaire that gathers information on the duration and the quantity of menstrual bleeding, the bother and inconvenience caused by the heavy blood loss, and the impact on daily activities. Questions posed are the following: (1) Do you experience menstrual bleeding for more than 7 days per month? (2) Do you experience 3 or more days of heavier menstrual bleeding during your menstrual period? (3) In general, does menstruation bother you due to its abundance? (4) During any of these heavier menstrual bleeding days, do you spot your clothes at night; or would you spot them if you did not use double protection/did not change your clothes during the night? (5) During these heavier menstrual days, are you worried about staining the chair, sofa, *etc.*? (6) In general, during these heavier menstrual bleeding days, do you avoid, as far as possible, some activities, trips, or leisure-time plans because you frequently need to change your tampon or sanitary towel? Affirmative answers to items 1 and 3 are assigned a score of 3 points each, whereas items 2, 4, 5, and 6 are assigned 1 point each. Negative answers are scored 0. The total score ranges from 0 to 10, with a score of ≥3 points being indicative of HMB. It is only validated in Spanish.^16^
- Two VAS measurements, one to assess the VASInt and the other to assess its VASImp. VAS scores were recorded by making a handwritten mark on a 100-mm horizontal line representing a continuum between “No bleeding at all” (scored 0) and “The heaviest possible bleeding I have ever seen” (scored 100) for VASInt and “It does not interfere with my daily activities at all” (scored 0) and “It totally interferes with my daily activities” (scored 100) for VASImp. Both were administered together in the same chart (Fig. 1).
- The EuroQoL five dimensions five levels questionnaire (EQ-5D-5L). The EQ-5D consists of a short descriptive system questionnaire and a VAS (EQ VAS). The EQ-5D-5L proposes five questions assessing five health dimensions (5D): mobility, self-care, usual activities, pain/discomfort, and anxiety/depression with five levels of severity (5L): no problems, slight problems, moderate problems, severe problems, unable to/extreme problems. Responses are rated from 1 (lowest degree of severity) to 5 (highest degree of severity). These are subsequently coded as single-digit numbers expressing the severity level selected in each dimension. The EQ VAS is a 200-mm vertical scale where the end points are labeled “The best health you can imagine” (scored 100) and “The worst health you can imagine” (scored 0). Patients are asked to mark their current state of health on the vertical scale.^19,20^ The validated Spanish version was used.^21^
- The Psychological General Well-Being Index (PGWBI), a questionnaire that comprises 22 polytomous items where a high score indicates high levels of psychological well-being. Six affective states are assessed within six subscales: anxiety, depressed mood, positive well-being, self-control, general health, and vitality.^22,23^ The validated Spanish version was used.^24^

### Outcomes

In the present analysis, we assessed the performance of the HMB-VAS as a screening tool using the PBAC as the reference standard and compared it with that of the SAMANTA questionnaire. Correlation with the PBAC and other QoL measurements was assessed.

### Statistical analyses

A multivariate discriminant analysis using a logistic regression model was carried out using clinically confirmed HMB versus control as the dependent variable and responses to the VASInt and the VASImp as independent variables. As for the SAMANTA questionnaire, the model took into account the time at which the PBAC test was performed (before or after the study visit) as a dichotomous variable to control for possible bias. A stepwise method was used to include the variables in the model. We analyzed the statistical significance of the logistic regression coefficients (β). The receiver operating characteristics (ROC) curve and the area under the curve (AUC) were calculated. A threefold cross-validation of the model was performed to further validate the model's performance.

For this purpose, the data set was randomly divided into *k* groups with the same proportion of values of the response variable (in our case, three). A single fold was used as validation data to test the model, and the remaining *k* − 1 folds were used as training data. This process was repeated *k* times with each fold used as validation data. The AUC for the ROC curve was estimated for this *k*-fold cross-validation and compared with the HMB-VAS values with the chi-square test.

The optimal cutoff value (Youden's index) was calculated. The accuracy, sensitivity, specificity, positive predictive value (PPV), and negative predictive value (NPV) of the VASInt and the VASImp scales (HMB-VAS) were calculated on the basis of this value. With the aim of simplifying the use of the HMB-VAS scale in clinical practice, a final model was generated using rounded coefficients (*i.e.*, natural numbers). The performance of this final model was tested to ensure the precision of the model.

Spearman's correlation coefficients were calculated to evaluate the convergent validity of the final HMB-VAS model using the EQ-5D-5L (index and VAS) and PGWBI questionnaires for reference.

No imputation of missing data was performed except for HMB case patients for whom a PBAC score was not available and not required due to medical decision and urgency of treatment. All analyses were performed using the SAS software, release 9.4 (SAS Institute, Inc., Cary, NC). Statistical significance was set at *p* < 0.05.

## Results

### Performance of the HMB-VAS screening tool

**FIG. 2.**
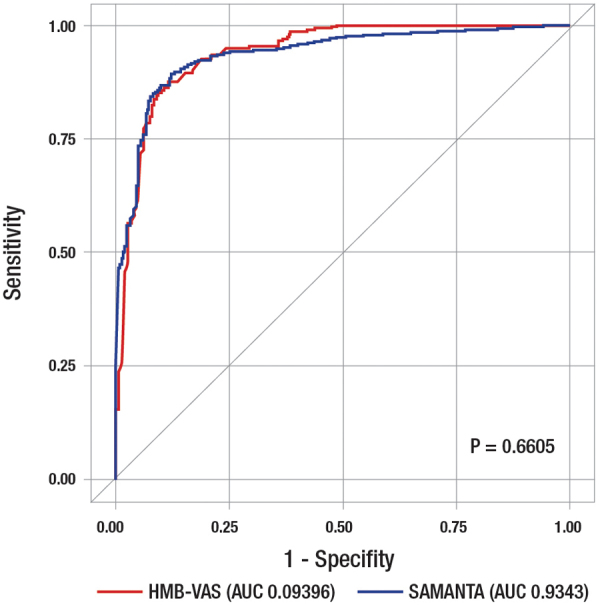
AUC for the HMB-VAS and SAMANTA questionnaire. HMB-VAS, final model including (VASInt + VASImp). AUC, area under the curve; VASInt, VAS measuring intensity of menstrual bleeding; VASImp, VAS measuring the impact of excessive menstrual blood loss on activities of daily living.

The resulting function was HMB-VAS score = 10.86 × VASInt score +2.48 × VASImp score. The accuracy, sensitivity, specificity, PPV, and NPV of this HMB-VAS screening tool are shown in Table 1. The sensitivity and NPV of the HMB-VAS were higher versus SAMANTA, but the specificity and PPV were lower, resulting in slightly lower accuracy (86.8% vs. 87.9% with the SAMANTA questionnaire). The AUC was 0.9396 for the HMB-VAS versus 0.9343 for the SAMANTA questionnaire (*p* = 0.6605) (Fig. 2). The AUC for the threefold validation model was 0.9388 (*p* = 0.5349 vs. SAMANTA). The cutoff point that maximized sensitivity and specificity in the model was 700, based on the calculation of Youden's index.

**Table 1.** Discriminant Capacity of the Heavy Menstrual Bleeding-Visual Analog Scale Tool (Raw and Final Model) Versus the SAMANTA Questionnaire

	Accuracy, %	Sensitivity, %	Specificity, %	PPV, %	NPV, %
HMB-VAS raw model	86.8	90.5	81.7	87.2	86.2
HMB-VAS final model	87.6	89.6	85.0	89.2	85.5
SAMANTA questionnaire	87.9	86.7	89.5	92.0	83.0

HMB, heavy menstrual bleeding; NPV, negative predictive value; PPV, positive predictive value; VAS, visual analog scale; VASImp, VAS measuring the impact of menstrual bleeding on daily life; VASInt, VAS measuring intensity of menstrual bleeding.

After rounding the regression coefficients (HMB-VAS score = 11 × VASInt score +2 × VASImp score), the performance of the HMB-VAS remained high, with 87.6% accuracy to detect HMB patients, using ≥700 as the cutoff score (Table 1).

### Correlation with other measurements

Spearman's rank correlation coefficients between the HMB-VAS, the PBAC, and the validated QoL PGWBI and EQ-5D-5L scales (VAS and index) are shown in Table 2. The correlation of HMB-VAS and the SAMANTA questionnaire was strong (Spearman's *r*: 0.79819) (*p* < 0.0001). The correlation with the PBAC was moderate to strong (0.59299; *p* < 0.0001). The correlation with the QoL (EQ-5D-5L) and well-being (PGWBI) scales was weak (−0.20332 and −0.24384 for the EQ-5D-5L VAS and Index, respectively, and −0.21680 for the PGWBI; all *p* < 0.0001) (Table 2).

**Table 2.** Spearman's Rank Correlation Coefficients Between Heavy Menstrual Bleeding-Visual Analog Scale and Pictorial Blood Loss Assessment Chart, the SAMANTA Questionnaire, VASInt, VASImp, and Quality-of-Life Scales

PBAC	SAMANTA questionnaire	VASInt	VASImp	PGWBI	EQ-5D-5L VAS	EQ-5D-5L index
0.59299	0.79819	0.99055	0.79657	−0.21680	−0.20332	−0.24384
<0.0001	<0.0001	<0.0001	<0.0001	<0.0001	<0.0001	<0.0001

HMB-VAS (final model including VASInt+VASImp with rounded coefficients).

PBAC, pictorial blood assessment chart; PGWBI, Psychological General Well-Being Index; VAS EQ-5D-5L, VAS of EuroQol five dimensions five levels questionnaire; VASInt, VAS measuring intensity of menstrual bleeding; VASImp, VAS measuring the impact of excessive menstrual blood loss on activities of daily living.

## Discussion

Our study shows the usefulness of the HMB-VAS as a tool for identifying women with HMB in the clinical setting, given that it offers similar performance to the validated HMB screening tool SAMANTA questionnaire. This result is consistent with the strong correlation found between both screening tools in the validation study of the SAMANTA questionnaire,^16^ and it is supported by the fact that both of them include the two fundamental aspects of the HMB definition,^3^ that is, the quantity of menstrual loss and its impact on activities of daily living (QoL).

The value of the HMB-VAS is especially interesting since this common and easy-to-use “graphic rating method”—which was first described in 1921^25^ and typically takes <1 minute to complete—may offer an alternative to the SAMANTA questionnaire in cases where literacy level or cultural background preclude using it. Both tools have advantages and disadvantages with respect to posing questions regarding specific problems and recording and interpreting the answers to these questions.

Conversely to the SAMANTA questionnaire, whose six questions are categorically scored as “yes–no,” the VAS makes it possible to express degrees for the responses to the questions posed.^26^ However, finding the point on the line that best fits one's feelings or attitudes may also be more difficult when these are not clear. The effect of socially desirable response behavior is likely to be lower for VAS than for other categorical psychometric scales, as it is more difficult to estimate the expected value. This translates into results that are closer to the respondents' true attitudes.^26,27^ VAS scales are considered to be “more accurate and sensitive and subject to less distortion and bias compared with categorical scales.”^26^

The reliability and validity of VAS measurements have been confirmed in a large number of studies. Moreover, these have been successfully used in real life for pain measurement and in other areas where a patient-reported score on a continuous scale is appropriate.^26^

The easiness of VAS to be understood, independent of literacy level,^17^ and to be culturally adapted with minimal translation difficulties to most frequently spoken languages^18,28^ means that it can be adopted in clinical practice when culturally validated versions of scales that go deeper into the problem (such as the SAMANTA questionnaire for HMB) are not available or are not suitable. It should be noted that variations in self-perceived feelings or attitudes (in our case with respect to what is considered “the heaviest possible bleeding I have ever seen” and “it totally interferes with my daily activities”) are likely to vary across cultures and literacy levels while being relatively homogeneous in groups with similar populations in these respects.

In our study, ∼90% of the study participants (in both the HMB and the non-HMB groups) had secondary or university studies, and a similar percentage was Spanish. It is also important to take into account that the VAS may be affected by age, especially when the impact on QoL is assessed. This is commonly perceived as higher in younger women.^27^ It would be interesting to perform an internal validation taking into account the above-mentioned aspects, as well as age and the burden of activities of daily living. Unfortunately, the homogeneity of our population with respect to the place of origin and literacy level did not allow this analysis.

It is worth noting that despite addressing the quantity of menstrual loss and the impact on activities of daily living (QoL), the HMB-VAS showed a moderate-to-strong correlation with the (semi)quantitative measurement method PBAC yet a weak correlation with QoL scales. This latter may be explained not only by a lower weighting of the impact of EML on daily life in the resulting model but also by differences in aspects measured by the EQ-5D-5L and PGWBI scales. Not in vain, the VASImp only focused on the interference of EML with activities of daily living. It would have been interesting to assess the correlation with the specific items measured by these two scales to get more insight into how the interference with activities of daily living translates into the QoL and well-being aspects assessed. In the interpretation of our results, it is important to take into account other limitations that are shared with the SAMANTA questionnaire.^16^ These include having used the PBAC as a reference, while this tool is known to progressively underestimate blood volume as menstrual loss increases^14^ and overestimate menstrual blood loss in the general community.^29^ Having used a threshold of 100 to discriminate between HMB or not has been questioned by some experts. However, we used the original version described by Higham et al., which established a threshold of 100 points above which HMB should be considered.^13^ The lack of homogeneity in demographic and clinical characteristics between HMB cases and controls, as a result of the consecutive manner in which patients were recruited should also be considering when interpreting the results.

Other limitations include the lack of homogeneity in the HMB diagnosis (PBAC vs. alternative ways, as described in the Materials and Methods section) and the moment when the PBAC was carried out (before the study or during the next menstruation), as a result of a wish not to disturb clinical practice. Nevertheless, this was overcome by including the timing of PBAC evaluation as a dichotomous variable in the model's developed.^5^ Another limitation was the lack of repeated PBAC, SAMANTA, and HMB-VAS measurements as it was an observational study based on a single visit. Well-designed studies are needed to confirm the usefulness of the HMB-VAS to screen for HMB in the clinical setting.

## Conclusion

The HMB-VAS shows good performance as a HMB screening tool, providing an easy-to-use alternative in special situations (*i.e.*, when the cultural background or literacy level preclude the use of other psychometric tools).

## Abbreviations Used

AUC: area under the curve
EML: excessive menstrual blood loss
EQ-5D-5L: EuroQoL five dimensions five levels questionnaire
FIGO: International Federation of Gynecologists and Obstetrics
HMB: heavy menstrual bleeding
NPV: negative predictive value
PBAC: pictorial blood loss assessment chart
PGWBI: Psychological General Well-Being Index
PPV: positive predictive value
QoL: quality of life
ROC: receiver operating characteristics
VAS: visual analog scale
